# Feasibility of Measuring Tobacco Smoke Air Pollution in Homes: Report from a Pilot Study

**DOI:** 10.3390/ijerph121214970

**Published:** 2015-11-30

**Authors:** Laura Rosen, David Zucker, Melbourne Hovell, Nili Brown, Amit Ram, Vicki Myers

**Affiliations:** 1 Department of Health Promotion, School of Public Health, Sackler Faculty of Medicine, Tel Aviv University, P.O. Box 39040, Tel Aviv 69978, Israel; nilibrown1970@yahoo.co.uk (N.B.); amit.bahar.ram@gmail.com (A.R.); vickim@post.tau.ac.il (V.M.); 2 Department of Statistics, Hebrew University, Mt. Scopus, Jerusalem 9190501, Israel; david.zucker@mail.huji.ac.il; 3 Center for Behavioral Epidemiology and Community Health, Graduate School of Public Health, San Diego State University, San Diego, CA 92123, USA; mhovell@cbeachsdsu.org

**Keywords:** children’s health, environmental exposure, tobacco smoke exposure (TSE), secondhand smoke (SHS), air quality (AQ), environmental tobacco smoke (ETS), respirable suspended particles (RSP), particulate matter (PM), environmental monitoring, smoke-free homes

## Abstract

Tobacco smoke air pollution (TSAP) measurement may persuade parents to adopt smoke-free homes and thereby reduce harm to children from tobacco smoke in the home. In a pilot study involving 29 smoking families, a Sidepak was used to continuously monitor home PM_2.5_ during an 8-h period, Sidepak and/or Dylos monitors provided real-time feedback, and passive nicotine monitors were used to measure home air nicotine for one week. Feedback was provided to participants in the context of motivational interviews. Home PM_2.5_ levels recorded by continuous monitoring were not well-accepted by participants because of the noise level. Also, graphs from continuous monitoring showed unexplained peaks, often associated with sources unrelated to indoor smoking, such as cooking, construction, or outdoor sources. This hampered delivery of a persuasive message about the relationship between home smoking and TSAP. By contrast, immediate real-time PM_2.5_ feedback (with Sidepak or Dylos monitor) was feasible and provided unambiguous information; the Dylos had the additional advantages of being more economical and quieter. Air nicotine sampling was complicated by the time-lag for feedback and questions regarding shelf-life. Improvement in the science of TSAP measurement in the home environment is needed to encourage and help maintain smoke-free homes and protect vulnerable children. Recent advances in the use of mobile devices for real-time feedback are promising and warrant further development, as do accurate methods for real-time air nicotine air monitoring.

## 1. Introduction

Exposure of infants and children to tobacco smoke is harmful and can be deadly, causing sudden infant death syndrome, respiratory illness, ear infections, exacerbating asthma, and contributing to delayed lung development in children [1]. While legislation has helped reduce exposure of the population in public places, forty percent of children worldwide are exposed to tobacco smoke in their homes [2]. This exposure has led to a large and entirely preventable burden of illness: worldwide in 2004, 166,000 child deaths and nearly 6 million child lower respiratory infections were attributed to secondhand smoke (SHS) [2].

Although there is a broad consensus about the need to protect children from tobacco smoke [1,3,4,5,6], there is no agreement about how best to do so. Convincing all household smokers to quit smoking and introducing a home smoking ban for visitors would be optimal. Interventions to encourage parents to quit smoking have shown limited benefit, with most parents continuing to smoke even within the limited duration of research studies [7,8], and expected relapse among many quitters [9]. As a result, there has been an increased focus on protecting children from tobacco smoke exposure (TSE) through the promotion of smoke-free homes and cars [6].

However, adopting a smoke-free home policy may be challenging, particularly since some smokers are heavily addicted to smoking tobacco. Further, they may be unaware of the level of tobacco smoke in their homes: this is especially true because most smoke is invisible [10] and many smokers have compromised ability to smell tobacco smoke.

As in other research fields dealing with environmental exposures, objective measures are available to determine the concentration of tobacco smoke in the environment. PM_2.5_, considered a “well-established marker for secondhand smoke” [11], measures the amount of small respirable particles less than 2.5 microns in diameter. It has been used extensively to assess secondhand smoke levels in hospitality venues and workplaces [12,13,14], as well as in homes [15,16,17,18,19,20] and cars [11]. Air nicotine has also been used to assess tobacco smoke pollution in the home. In addition to being sensitive to tobacco smoke, air nicotine is a specific indicator of tobacco smoke pollution, unlike PM_2.5_. This is the primary reason why the US Surgeon General prefers air nicotine over PM_2.5_ [1].

In order to convince parents to maintain smoke-free homes, some investigators have tested home air quality and provided the parents with the evidence. Investigators have also used home air quality to evaluate the effectiveness of interventions to reduce tobacco smoke in the home.

We developed an intervention program, Project Zero Exposure [21], which aimed to decrease exposure of young children to tobacco smoke, and conducted a pilot study of our program in 2013–2014. In the context of the pilot, we measured both PM_2.5_ and air nicotine. We were interested in assessing the feasibility of measuring PM_2.5_ and air nicotine in a real-world setting, and in assessing whether delivering objective feedback to parents could help them internalize how their smoking affects air quality, and therefore change their behavior. We also wanted to obtain estimates of the effectiveness of the intervention as measured by changes in PM and air nicotine, in order to design a randomized controlled trial to test the intervention’s effectiveness. Obstacles surrounding air quality measurement came to our attention during the baseline measurement period. In this paper, we describe our experiences with measurement of tobacco smoke air pollution in the homes of participants.

## 2. Experimental Section

### 2.1. Ethics and Trial Registration

We received approval for the trial from the Tel Aviv University Ethics Committee, the Asaf HaRofe Hospital Helsinki Committee, and the Ministry of Health Helsinki Committee. We also received approval for recruitment from the National Educational Supervisor of Naamat daycare centers. Written informed consent was obtained from all participants during the first home visit. The trial is registered in the NIH Clinical Trials Registry, NCT01335178.

### 2.2. Design

This pilot study used a one-group, before-and-after design to assess the acceptability and feasibility of an intervention designed to help parents in smoking families protect their children from tobacco smoke, and to estimate the expected changes in outcome variables. The sample size of the pilot study was based on logistical and financial considerations.

### 2.3. Participants

Parents from families where smoking occurred were recruited to the study between March and September 2013. Eligibility criteria were: having a child of age 8 or less, at least one smoking parent in the family, and willingness to join the study. Parents were recruited via child daycare centers. Interested parents received a flyer about the program, and provided contact information to project staff. Once a parent agreed to participate, the spouse/partner was invited to participate as well. Participating families received a gift certificate worth NIS 250 (about $60) as compensation for their time.

### 2.4. Intervention

The intervention consisted of the following elements: (a) Three motivational interviews, scheduled for baseline, one month, and three months; (b) Feedback on air quality in the home (from a Sidepak and/or a Dylos monitoring device, and from passive air nicotine dosimeters); (c) Feedback on child’s exposure via hair samples analyzed for nicotine; (d) a website designed especially for the project [22];and (e) various self-help materials, including a booklet, a magnet about TSE, and air fresheners.

The goal of the motivational interviews was to encourage the parents to decide to change their smoking behavior to protect their children from exposure to tobacco smoke. During the motivational interviews, parents were shown graphs obtained from a Sidepak or Dylos monitor, and were informed of the level of exposure in relation to the acceptable level of air quality as reported by the World Health Organization (0.025 mg/m^3^) [23]. Results from air nicotine and hair nicotine measurements were given to the parents once they became available.

### 2.5. Baseline Data Collection

An initial visit was made to all participants to obtain informed consent and parentally reported information on demographics, smoking behaviors in the home, and child characteristics. At that time, an air nicotine dosimeter was placed in a central area in the home and remained there for a period of 7 days. During a second visit six days after the first one, the Sidepak was placed in a central area in the home for a period of 8 h. During that period, participants could easily see the Sidepak readings, which were clearly displayed on the monitor, and showed per-second levels of PM_2.5_ values. The following day, a motivational interview was held which included downloading results from the previous evening’s Sidepak monitoring, and graphical presentation of the results on the interviewer’s computer.

### 2.6. Particulate Matter (PM) Measurement

We measured PM_2.5_ using a Sidepak with all families, and using a Dylos Monitor in some families. The TSI SidePak AM510 Personal Aerosol Monitor (TSI, Inc., St Paul, MI, USA), which measures RSPs less than 2.5 µm diameter (PM_2.5_), uses light scattering to determine mass concentration of particles. A built-in sampling pump draws air through the device, which then measures the real-time PM_2.5_ concentration in milligrams per cubic meter. Before each data collection session the SidePak was charged, cleaned, and zero-calibrated using the HEPA filter and the standard calibration procedure according to the manufacturer’s specifications.

### 2.7. Original Protocol for PM Measurement

The original protocol called for use of a Sidepak at the baseline, 1-month post-baseline, and 3-month post-baseline visits. We intended to use the Sidepak for a 24-h reading prior to each of these visits, and to use the Dylos as needed, depending on logistical considerations, as the monitors were left in the homes for a 24-h period and only two Sidepaks were available.

### 2.8. Measurement of Air Nicotine

Air nicotine was measured using passive nicotine dosimeters in central rooms in the homes of the participants for a period of approximately 7 days. The first assessment was done just prior to the first motivational interview. The exact number of days of exposure was considered when calculating exposure. After use, the monitors were stored at −20 degrees (°C) and shipped frozen to Mass Spectrometry Services at the San Diego State University.

### 2.9. Description of Findings

We first present a table with the mean, minimum, and maximum PM_2.5_ level at the baseline visits. We then describe our experience with the PM measurements by presenting selected graphs from continuous 8-h PM monitoring sessions. The graphs were chosen to demonstrate common scenarios encountered. We report on notes taken by our staff about challenges in using these graphs to present information about smoking in the home to parents, and detail the changes which we made to the original protocol in light of our experiences. We present a graph from real-time PM monitoring with the Sidepak, show a graph comparing continuous PM measurement using the Sidepak and the Dylos Monitors, and discuss our experience with use of passive dosimeters for air nicotine measurement.

## 3. Results and Discussion

### 3.1. Results

#### 3.1.1. Participants

Seventy-four parents were willing to receive information about the program. Of these, 29 families consented to join the project. Twenty participants were smokers, while nine were partners of smokers. In seven families, both parents participated. Two families did not provide Sidepak data, leaving 27 families with baseline data. The mean age of the children was 3.4 (±2.2) years (range 9 months–8.5 years); 12 were male and 16 female.

#### 3.1.2. Descriptive PM Statistics

Table 1 presents the mean, minimum, and maximum PM_2.5_ readings from the baseline recording session. The mean PM values varied from 0.004 μg/m^3^ to 0.03 μg/m^3^ (group mean 0.022 ± 0.036 μg/m^3^; group median 0.0165 μg/m^3^), and the highest recorded value was 4.641 μg/m^3^.

**Table 1:** Mean, minimum and maximum PM_2.5_ readings for each participating family at baseline (μg/m^3^).

Family	Mean	Minimum	Maximum
N1	0.009	0.001	0.126
N2 mother	0.008	0.002	0.414
N2 father	0.018	0.006	0.441
N3	0.013	0.004	0.171
N4	0.018	0.008	0.356
N5	0.021	0.006	0.214
N6	0.016	0.005	0.389
N7	0.200	0.008	1.471
N8	0.015	0.003	0.293
N9	0.007	0.000	0.172
N11	0.027	0.002	0.375
A1	0.017	0.007	0.364
A2	0.024	0.007	0.488
A3	0.006	0.000	0.467
A4	0.030	0.004	0.528
A5	0.019	0.008	0.245
A6	0.020	0.000	1.418
A7	0.013	0.005	0.182
A8	0.022	0.008	0.640
V1	0.009	0.003	0.166
V2	0.005	0.000	0.297
V3	0.009	0.001	0.375
Y1	0.015	0.004	0.525
Y2	0.030	0.0100	0.188
Y3	0.008	0.002	0.320
Y4	0.020	0.001	4.641
Y5	0.004	0.000	0.783
Y6	0.018	0.007	0.364

#### 3.1.3. Results of Continuous 8-h PM_2.5_ Monitoring Using the Sidepak

**Figure 1:**
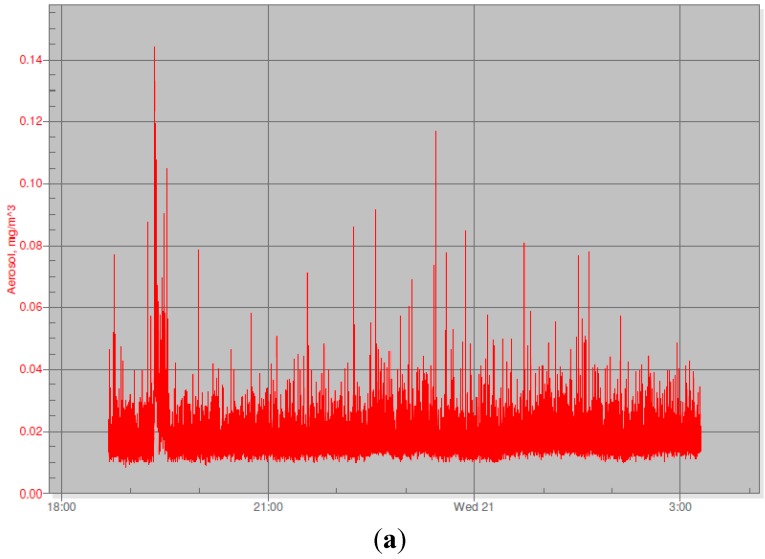

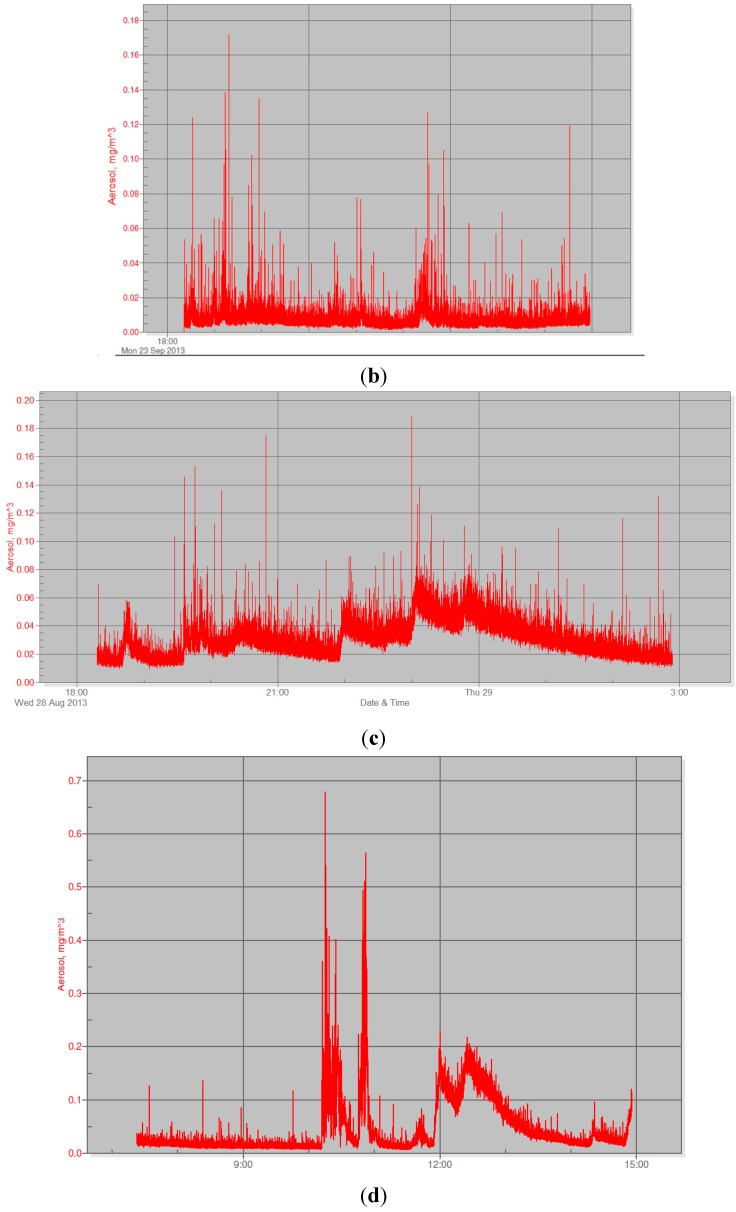

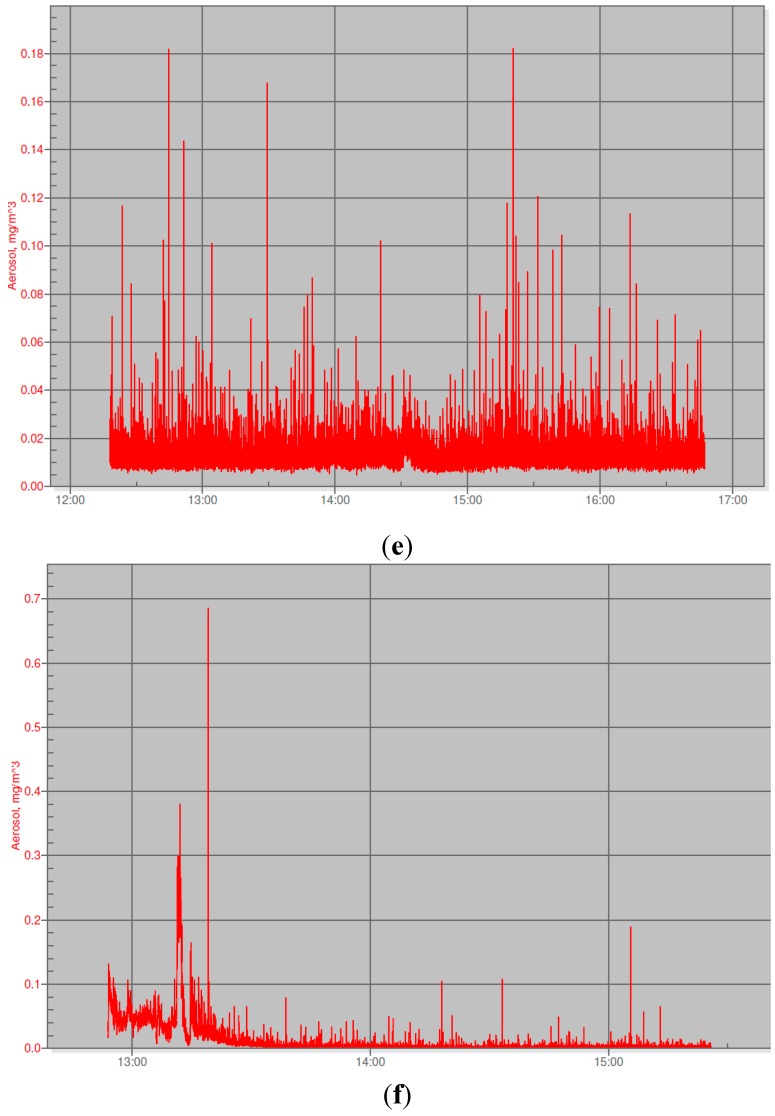
(**a**) Continuous monitoring of PM with a Sidepak from 18:00 to 03:00 the following day—no smoking reported; (**b**) Continuous monitoring of PM with a Sidepak—no smoking reported, bread baking occurred; (**c**) Continuous monitoring of PM with a Sidepak—smoking in the home; (**d**) Continuous monitoring of PM with a Sidepak—smoking in the home; (**e**) Continuous monitoring of PM with a Sidepak—outdoor air pollution; (**f**) Continuous monitoring of PM with a Sidepak—no smoking, outdoor air pollution.

Graphs of Sidepak PM readings from six of the homes are presented in Figure 1a–f. Figure 1a shows a fairly constant level of exposure with many small peaks and a more obvious peak around 19:30, indicating a short-term increase in PM which quickly returned to background level. This is suggestive of a cigarette having been smoked; however, participants reported that no one smoked during the whole evening of recording. The 19:30 peak may have been caused by cooking around that time. The interviewer found this graph unhelpful in the context of the intervention.

Figure 1b shows continuous background exposure at fairly low levels, with several noticeable peaks during the evening. Participants reported that there was no smoking at all during the time of recording, but said that bread was being baked at the time. Both Figure 1a,b show peaks as high as about 140 and 170 μg/m^3^. Figure 1c is much more dramatic, showing exposure levels of up to about 190 in the home of a family where both parents smoke regularly on the closed-in balcony. In contrast to the previous examples, this graph demonstrated clearly to the family the effects of smoking in the home. The parents agreed that the peaks were caused by cigarette smoking. Figure 1d shows a very high level of exposure —nearly 700—from smoking in the home; the interviewer found the graph helpful during the motivational interview. Figure 1e shows an example of a “noisy” graph from the Sidepak. According to participants, no smoking occurred except for the small peak around 14:30 when a cigarette was smoked. In contrast, the high level of particles seen for the rest of the measurement period most likely represent air pollution. This presented a problem for the interviewer: the participant was convinced that high levels were bad, and so considered the lower levels caused by smoking relative to other sources of RSPs as less damaging. This compromised the Sidepak’s potential to convincingly demonstrate tobacco smoke exposure. Figure 1f shows results from a household in which no smoking at all was reported during the measurement period. At the start of the period, the windows were open, and there was a dust storm outside. Later the windows were closed, and the levels of particles fell. This again presented a problem for the interviewer, as it seemed to corroborate the smoker’s beliefs that outdoor air pollution presented a greater risk than smoking. 

The much higher PM_2.5_ values reportedly caused by non-smoking sources caused some parents to erroneously conclude that tobacco smoke was not really dangerous, since the PM_2.5_ levels were sometimes lower with tobacco smoke than with everyday occurrences such as dust or burning food. Additionally, we received many complaints about the noise levels of the Sidepak, which were turned on for 8-h periods.

#### 3.1.4. Real-Time Monitoring with the Sidepak

On discovering the problems with continuous monitoring of PM_2.5_ with a Sidepak, we experimented with real-time testing, measuring PM_2.5_ while participants lit up and smoked a cigarette in the vicinity of the machine. The changes in PM_2.5_ levels were recorded for several minutes, and watched in real-time, as well as being uploaded to the interviewer’s computer and shown to the participant.

**Figure 2:**
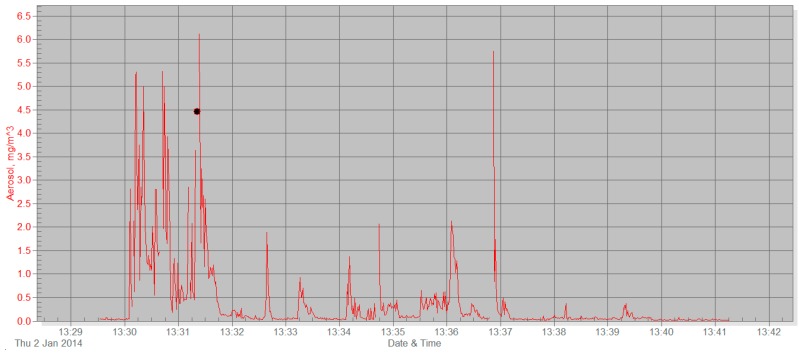
Real-time PM monitoring with Sidepak—two smokers outdoors.

Real-time measurements produced clear graphs, where the effect of the cigarette was much more obvious. When real-time monitoring was used for a much shorter period of time (10–15 min, not 8 h), it was far easier to discriminate the immediate effect of cigarette smoke on the level of PM_2.5_ in the air. Figure 2 shows results from just 15 min of monitoring. Two people were smoking outside, near the Sidepak, one after the other. PM_2.5_ increased substantially after the smokers lit up.

#### 3.1.5. Use of the Dylos Monitor

Because of difficulties with the Sidepak, we also tested a different PM monitor, the Dylos (DC1100 Air Quality Monitor), which is a laser particle counter that allows real-time monitoring of indoor air quality. We conducted a simultaneous real-time smoking test in a participant’s home using both the Sidepak and Dylos, over a 25 min period, in order to compare both for accuracy and for representation.

**Figure 3:**
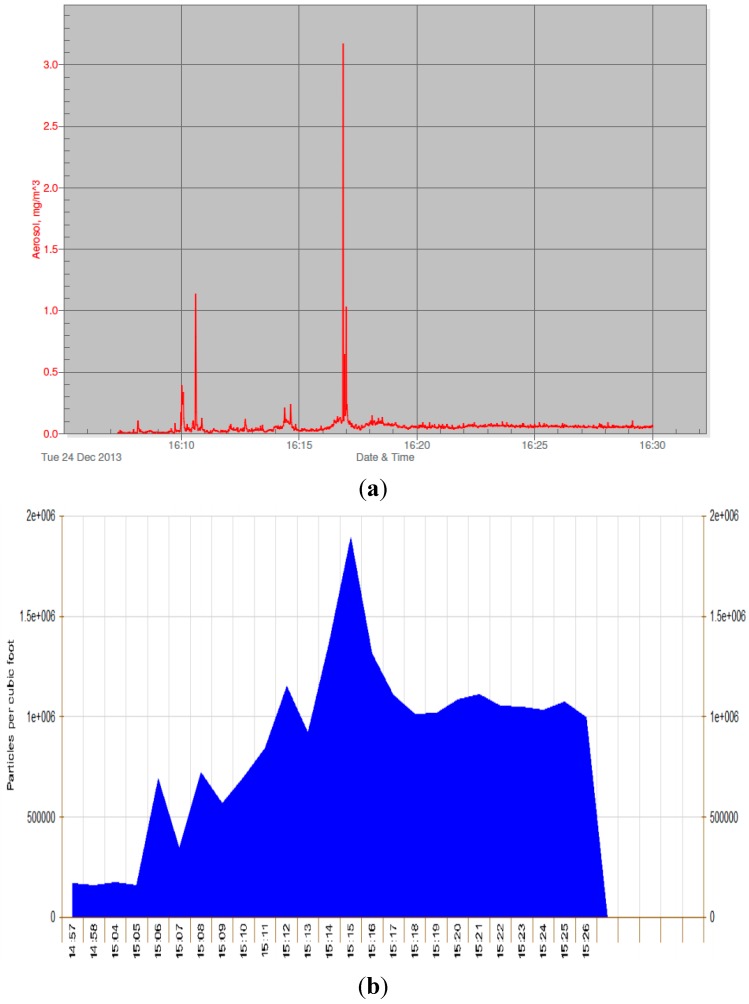
Real-time PM monitoring—simultaneous test with (**a**) Sidepak and (**b**) Dylos.

Figure 3 shows the results from that test (Note: the time was not set correctly on the Dylos machine). Results are similar, although the Dylos graph is more impressive and potentially easier to comprehend. The Dylos had the advantage of being quieter and cheaper than the Sidepak.

#### 3.1.6. Air Nicotine Measurement

During the study, questions arose relating to the shelf life of the dosimeters, and whether dosimeters stored for several (up to 6) months prior to use are as effective or would produce identical results to those recently manufactured, and whether temperature during storage and transport would affect results. Our dosimeters were kept in a freezer as recommended for optimal conditions. Different labs provided different instructions on whether monitors needed to be shipped frozen to maintain accurate results, on shelf life, and on the optimal time period for measuring exposure (1 week or 2 weeks). At baseline, the levels of air nicotine ranged from 0 to 1.1753 ng/mg, with a mean of 0.1159 ± 0.253 (*n* = 29). The median was 0.0194 and the interquartile range was 0.09765. High levels of uncertainty were reported by the laboratory at very low levels of exposure.

### 3.2. Discussion

This pilot study demonstrated the elusive nature of accurately measuring tobacco smoke air pollution in the home, and using measurements to persuade parents to protect children from tobacco smoke in the home. Continuous monitoring of home PM_2.5_ with the Sidepak yielded results which were often inconsistent with reported times of tobacco smoking in the home, and appeared to be highly affected by non-tobacco sources such as cooking, heating and environmental exposures. The variability of the graphs of PM levels from continuous monitoring, which included a great deal of minute-to-minute variability, unexplained peaks, and high PM_2.5_ levels which were apparently due to cooking and outdoor pollution, led to confusion in interpretation for interviewers and participants alike. In some cases, levels of non-tobacco RSP sources were substantially higher than from tobacco sources, according to participant reports. In those cases, the lower PM_2.5_ associated with tobacco smoke relative to environmental factors may have led participants to incorrectly believe that tobacco smoke was less damaging than other sources of air pollution. After convincing parents that the levels of PM_2.5_ were important, it was difficult to explain that the content of PM_2.5_, and not the amount, was of paramount importance. We also received complaints about the noise levels of the Sidepak.

Previous reports in the literature support our finding that other sources of PM_2.5_ may interfere with PM_2.5_ readings when used to measure tobacco smoke exposure. For example, a study investigating the effect of a smoking ban in a prison mentioned that the Sidepak, left recording for 3 months, was situated next to a toaster and microwave which may have interfered with results [24]. Studies under laboratory conditions—controlled chamber experiments—showed that toasting bread generated higher peak concentration (1.0 *vs*. 0.30 mg/m^3^) and higher emission rate (4.2 *vs*. 2.8 mg/min) than cigarette smoke [25]. Another study reported that although Sidepak monitors are factory calibrated to 1.0, for test dust, calibration factors in fact varied for different emission sources, from 0.32 for cigarette smoke, to 0.70 for frying a hamburger [26]. The highest emissions of PM_2.5_ in that study came from burned foods (15 mg/min^−1^) and fireplaces (16 mg/min^−1^), which were much higher than cigarette emissions (3.8 mg/min^−1^).

By contrast, the Scottish REFRESH study [20] used 24-hour continuous PM_2.5_ monitoring without reports of problems. Respondents in that study reported that they were “shocked” to see the high PM_2.5_ levels which resulted from their smoking. Homes with open coal, wood, or peat fires were excluded from that study. Differences in home architecture may have contributed to better results: in Israel, open kitchens are common, most people live in urban areas, and windows are often open due to the temperate weather. These elements may have contributed to the questionable persuasiveness of 8-hour continuous PM monitoring in our study as compared with the REFRESH study.

Real-time PM monitoring was found to be a feasible means of demonstrating tobacco air pollution, as the increase in RSPs was immediately seen when the smoker was in the vicinity of the monitor. Participants reacted more positively to these results, finding them easier to understand. Our real-time PM monitoring procedure was similar to the procedure followed in Harutyunyan’s study: indoor and outdoor PM_2.5_ levels were measured using a Sidepak, participants lit their cigarettes indoors while the Sidepak was operating, and the data were immediately uploaded to a computer in order to provide “risk-based personalized feedback” [17].

The real-time results from both the Sidepak and the Dylos monitor were well-received by participants. The main advantage of the Dylos is the cost: the initial purchase cost is about one-tenth of that of the Sidepak (Sidepak: $4100, Dylos: $425) [27], and there is no need for costly annual calibration by the manufacturer as there is with the Sidepak. The major advantage of the Sidepak is that it provides information in the same units used by the EPA for air quality (PM_2.5_ μg/m^3^), and so the numbers are directly interpretable to those aware of EPA’s Air Quality Index. The Dylos reports number of particles per 0.01 cubic foot, which then can be converted to the better-known PM_2.5_. The Dylos is much quieter. The cost and noise level factors suggest that Dylos is a more feasible option than the Sidepak for investigators and those interested in real-time home air quality.

PM_2.5_ measurement is a commonly-used method for assessing tobacco smoke air pollution in hospitality venues. In a study of 128 Irish pubs in 15 countries, the PM_2.5_ levels were 93% lower in non-smoking venues than levels in smoking venues [12]. Often in hospitality venues many people smoke at once and food preparation takes place in a separate area. The home environment likely has a very different mix of tobacco smoke contamination and other sources of contamination. For this reason, although assessment of tobacco smoke air pollution using PM_2.5_ levels may be appropriate for hospitality venues, its use in the home environment may depend on specifics of climate, congestion, and architecture.

We tested the use of passive air nicotine monitors, which are both sensitive to and specific to tobacco smoke. We were unable to find a good solution to the logistic issue of providing timely feedback: analyses had to be done in bulk, and this meant waiting till all baseline samples were collected. It took several months to obtain results, and by then the participants did not recall particulars of smoking in the home at the time of the measurement. We were also challenged by the uncertainty regarding the shelf-life of the dosimeters, the need for frozen storage and or transport, and the optimal length of measurement time. The literature is inconsistent on these matters, with measured length of time ranging from one week [15,16,28,29], to 2 weeks [30], to 6 months [18]. Further, there was concern that the nicotine dosimeters may have been contaminated during storage or transport.

The complexities of PM and air nicotine monitoring suggest that the scientific basis for tobacco air pollution measurement in the home environment needs to be strengthened. Objective feedback to parents to encourage smoke-free homes, evaluation of interventions, and population monitoring would all benefit from a clearer scientific basis for monitoring in the home. Immediate feedback is of particular importance for delivering a clear message to parents: the potential for decreased intervention efficacy due to delayed feedback has been previously reported in the context of behavior change [31].

Several interesting advances in the field have been reported recently. Use of sounds and lights in the home based on changes in PM levels, is one promising approach to encourage behavioral change: the sounds and lights provide immediate feedback in an ongoing manner, are acceptable to most participants, and allow residents to immediately discriminate between changes in PM levels caused by cooking or dust, and changes due to tobacco use [32,33,34]. Another interesting solution is a real-time nicotine detector which is currently being developed for commercial use. It provides sensitive, specific, and immediate information [35]. The model under development automatically adjusts to baseline ambient nicotine levels, and detects changes in air levels, allowing immediate notification if someone lights up a cigarette.

However, even these more sophisticated feedback processes remain underspecified with respect to Principles of Behavior. Although simple (and sometimes complex) feedback can serve as a punitive or reinforcing consequence that alters behavior, these functions are not guaranteed. Smokers are very much aware that they are smoking and do not need any other special measure to be assured that they are smoking. Feedback of the sort tested here takes place in a context where investigators are using it to encourage reduction in home smoking or a complete ban on smoking in the home and thereby protect the health of children exposed to SHS. The incentive of protecting children or of pleasing clinicians or investigators provides far greater theoretical motivation for change than simple feedback. Tobacco addiction is one of the most powerful addictions known, and thus the incentive of pleasing a provider or investigator is unlikely to reliably curb the addiction, but the incentive of protecting children may do so. More research is needed to untangle these issues. It seems likely that future studies will require stronger reinforcement strategies for promoting total cessation of smoking, or not smoking in the home. These strategies might be made even stronger by linking them to real-time objective measures of smoke in the home. Our study shows that consumer satisfaction with the Sidepack and Dylos instruments needs to be refined to open the way to testing more powerful reinforcement strategies that can be delivered with precision only with real-time, time series data.

Finally, home indoor environments pose a pulmonary health risk to children when they are exposed to other sources of smoke, for example, burning food or fireplaces, or dust. All these exposures may involve a host of toxic agents. In order to fully protect children, precise measures of agent-specific sources of contamination are needed for tobacco as well as other toxins.

## 4. Conclusions

The science of home air quality measurement for tobacco smoke exposure needs to be strengthened and standardized in order to make it possible to provide persuasive evidence to parents which is specific to tobacco smoke air pollution, to evaluate interventions, and to monitor population levels of pollution. PM_2.5_ is useful as a real-time feedback mechanism, and the low cost and noise level of the Dylos device make it a realistic option for even large-scale investigations. The appropriateness of PM_2.5_ for continuous monitoring or assessment of tobacco pollution levels in the home environment may be compromised by environmental conditions such as climate and urban life. Logistic factors relating to air nicotine measurement, such as time needed to leave the monitor in place, shelf-life, storage and transport conditions should be carefully evaluated and standardized. Resources to improve the science of addressing tobacco smoke air pollution in the home environment are warranted, and should focus on feasible, sensitive, specific, and cost-conscious approaches that place due emphasis on the fact that smoking poses a considerable health risk without any health benefits. Tobacco-related disease warrants investment in real-time measures that can be employed to curtail smoking in homes, other micro-environments and around children. Real-time measurement of air nicotine and use of mobile devices for measurement of air nicotine or PM, with appropriate behavioral strategies, are particularly promising.
